# Experimental Evolution as a High-Throughput Screen for Genetic Adaptations

**DOI:** 10.1128/mSphere.00121-18

**Published:** 2018-05-09

**Authors:** Vaughn S. Cooper

**Affiliations:** a Department of Microbiology and Molecular Genetics, University of Pittsburgh School of Medicine, Pittsburgh, Pennsylvania, USA; b Pittsburgh Center for Evolutionary Biology and Medicine, University of Pittsburgh School of Medicine, Pittsburgh, Pennsylvania, USA; Escola Paulista de Medicina, Universidade Federal de São Paulo

**Keywords:** evolutionary biology, genomics, population genetics

## Abstract

Experimental evolution is a method in which populations of organisms, often microbes, are founded by one or more ancestors of known genotype and then propagated under controlled conditions to study the evolutionary process. These evolving populations are influenced by all population genetic forces, including selection, mutation, drift, and recombination, and the relative contributions of these forces may be seen as mysterious. Here, I describe why the outcomes of experimental evolution should be viewed with greater certainty because the force of selection typically dominates. Importantly, any mutant rising rapidly to high frequency in large populations must have acquired adaptive traits in the selective environment. Sequencing the genomes of these mutants can identify genes or pathways that contribute to an adaptation. I review the logic and simple mathematics why this evolve-and-resequence approach is a powerful way to find the mutations or mutation combinations that best increase fitness in any new environment.

## Abstract (precis)

Experimental evolution is a method in which populations of organisms, often microbes, are founded by one or more ancestors of known genotype and then propagated under controlled conditions to study the evolutionary process. These evolving populations are influenced by all population genetic forces, including selection, mutation, drift, and recombination, and the relative contributions of these forces may be seen as mysterious.

## OPINION/HYPOTHESIS

Experimental evolution is a method that is gaining in popularity because of several inspiring successes and because high-throughput sequencing can reveal the genetic basis of the evolutionary process (1, 2). An underappreciated benefit of these studies is their ability to identify mutants in genetic pathways that underlie functions of interest. Thus, the experimental evolution of large populations of microbes can and should be viewed as a powerful genetic screen for adaptations. I outline why the processes underlying most evolution experiments are practically straightforward and why evolved mutants can provide valuable insight into the biology of the microbe under study and its response to environmental pressures.

Experimental evolution is not a new method, dating back to the 1870s when Dallinger conducted continuous culture experiments at steadily increasing temperatures (3). It also still underlies a large fraction of vaccine development, in which viruses are propagated in alternative hosts or cell lines to render them nonpathogenic but still immunogenic (4). Likely the best known is the Long-Term Evolution Experiment (LTEE) in which 12 Escherichia coli populations have been propagated daily for more than 30 years (5). Many remarkable studies of the LTEE have taught us a great deal about the evolutionary dynamics of adaptation and revealed numerous adaptive phenotypes, including gains in cell size, metabolic capacity, thermal tolerance, life history parameters, and above all, competitive fitness. Yet for the first 13 years of the LTEE, the genetic sources of adaptation remained unknown because the causative mutations did not affect the candidates chosen for Sanger sequencing. The first few mutations were discovered with higher-throughput screens of metabolism, expression microarrays, and by probes targeted to mobile insertion sequences (6–8). The power of each of these methods now pales in comparison to today’s genome-enabled screens that harness simple population genetic processes to precisely identify genetic adaptations.

Evolution experiments are influenced by all population genetic forces: selection, genetic drift, mutation, and recombination. In conducting a genetic screen, we can usually ignore the process of recombination, given that we are mostly interested in “first-step” adaptive mutants arising in different clones. Given that mutations occur inevitably, the relative balance of the remaining forces is governed by the effective population size, *N*_*e*_, and the strength of selection, *s*, acting on these mutations (Table 1). *N*_*e*_ is a property that is typically much less than the census population size (*N*) at any given time and is biased toward bottlenecks that occur during transfers. In practice, microbial populations are transferred at sizes ranging from a single cell to ~10^8^ individuals or more. Meanwhile, values for *s* that have been measured during experimental evolution range from ~0.01 (e.g., a minor improvement in resource uptake [6]) to ~4 (9) (involving the absolute gain in ability to colonize a new niche), with smaller values more common. Mutants with *s* < 0.01 are likely much more abundant but remain largely undetected because they are lost by drift or outcompeted by more beneficial mutants in large experimental populations, a process known as clonal interference (Table 1) (2, 10). However, technologies that rarify cells and limit clonal interference could detect even smaller fitness effects (13, 42).

**TABLE 1:** Key terms used in this article

Term	Definition
Effective population size (*N*_*e*_)	This is the size of an ideal population, in which all individuals reproduce equally and experience no fluctuation in size, which experiences the same genetic drift as the actual population. It is typically calculated as the harmonic mean of the population sizes at the time of transfer and the fraction that is transferred, which is strongly biased toward the bottleneck size.
Selective coefficient (*s*)	The fitness difference between a given genotype and typically, a wild-type genotype, in units of time^−1^. Commonly in experimental evolution, *s* is defined as the difference in Malthusian parameters between the mutant and wild type as follows: ln (*N*_*m*_1/*N*_*m*_0) − ln (*N*_WT_1/*N*_WT_0), where *N* is cell number and *m* is mutant and WT is wild type at time 0 or 1 (e.g., *N*_*m*_1 is the number of mutant cells at time 1).
Establishment	The process whereby a mutation rises to a high enough frequency to escape loss by drift, i.e., greater than experimental population bottlenecks and greater than 1/*s*.
Clonal interference	Competition between beneficial mutants in an asexual population resulting in the loss of less beneficial mutants and the delayed rate of fixation of the most beneficial mutant (10). Also known as the Hill-Robertson effect in sexual populations, where the process may be overcome by recombination that assembles beneficial mutations within the same genome (41).

**FIG 1:**
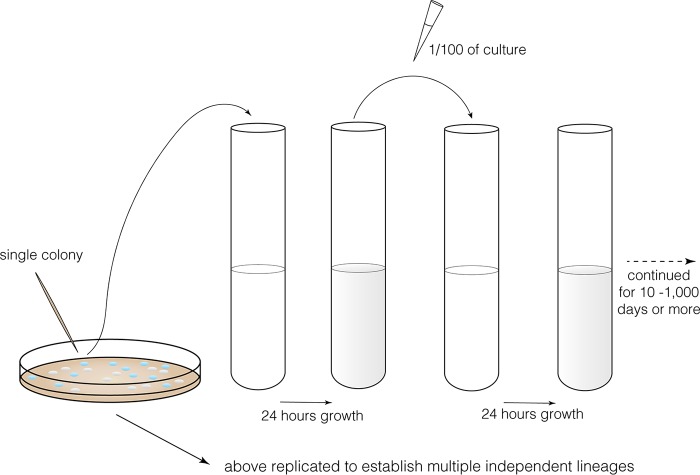
Design of a typical evolution experiment with microbial populations. A single colony is isolated from an agar plate and used to inoculate a tube of growth medium. Every 24 h for days or weeks, 1/100 of the population is transferred to fresh medium enabling regrowth. Multiple replicate populations are typically established by the same protocol and can be tracked by plating, quantitation of genetic markers, or whole-population genomic sequencing.

When evolving populations are maintained under conditions in which the product of these two properties *N*_*e*_ and *s* is clearly greater than 1 (*N_e_s* ≫ 1), then selection becomes the dominant force in the population (2, 21, 43). To understand these properties more practically, consider a typical serial dilution evolution experiment in which bacterial populations are grown from a single clone in medium that supports a population of 10^8^ cells/ml (Fig. 1). Let us also assume that the ancestor is not preadapted to this environment so there is opportunity for improvement. Population growth on the first day involves ~10^8^ cell divisions and generates ~10^5^ mutations given the approximate per-genome, per-generation mutation rate of 10^−3^ (11, 12). Selection could act upon any of these ~10^5^ mutations, and the probability that any one of them would reach a detectable frequency (say, 0.01) depends on the mutant’s frequency and its selective value. So, the earlier a mutation occurs on this first day of population expansion, the more likely it will reach high frequency. Yet most mutations arise in the final division of population growth, i.e., when the population increases from 5 × 10^7^ to 10^8^ cells/ml, involving 5 × 10^7^ cell divisions. The key point is that after this first growth cycle, there are already many mutations present in the population, but nearly all are very rare, having arisen in the last generation (16).

In a side note, in considering these estimates, one may wonder if some sites or genome regions may be more mutation-prone than others, thus increasing the number of certain mutations. While mutation rates do vary among sites, such rate variation typically involves fold changes (2× to 5×) and rarely more than an order of magnitude (10×) (11, 12). These processes can be influential over hundreds of generations but typically contribute little over short time scales because they would add only a handful of new mutations to a sample of ~10^5^. Nonetheless, if these mutation hot spots encode a trait under strong selection, such as phase variation of membranes affecting antibiotic resistance (14, 17, 18), they may greatly accelerate the evolutionary rate.

On the next day of the experiment, we transfer some fraction of the first population to fresh medium, say 1/100, and growth resumes (Fig. 1). For example, 10^6^ cells/ml harboring ~10^3^ mutations (simple dilution) now regrow to reach 10^8^ cells/ml, which involves another 10^8^ cell divisions (10^6^ divisions to reach 2 × 10^6^ cells/ml, 2 × 10^6^ divisions to reach 4 × 10^6 ^cells/ml, and so on) and produces another 10^5^ mutations. Despite the many individual cell divisions, it is important to realize that the number of generations the entire population undergoes during the next cycle is log_2 _(10^8^/10^6^) = 6.67 generations. This is the number we commonly reference in describing the duration of an evolution experiment over any interval. It is also noteworthy that this demographic measure of generation number does not depend on the population size but rather on the dilution itself. Under these conditions, the *N*_*e*_ of a population cycling between 10^6^ and 10^8^ cells/ml each day is ~2 × 10^6^ (Table 1).

**FIG 2:**
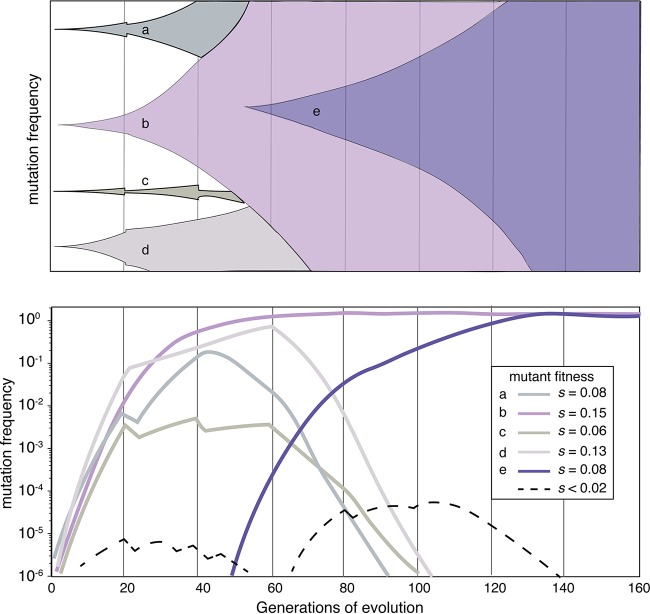
Two representations of the same mutation frequency dynamics during a hypothetical evolution experiment. (Top) Muller plot demonstrating the establishment and rise of four adaptive lineages and eventual fixation of the most-fit competitor (mutant lineage b), shown in purple. A second mutation (mutant lineage e) that further increases fitness subsequently fixes within the purple lineage. (Bottom) Plot of log mutation frequency over time, which demonstrates that adaptive lineages must rise in frequency by several orders of magnitude before they may be easily detected. All lineages may be influenced by transfer bottlenecks that randomly increase or decrease their frequency (see erratic changes in the lineages that went extinct). Dashed lines denote mutations that did not reach a conventionally detected frequency and were driven extinct by competition with more-fit lineages. Dynamics are based on simulations generated by FFPopSim (40) with asexual lineages and periodic 1:100 population bottlenecks.

Now, revisiting the condition in which the force of selection dominates, *N_e_s* ≫ 1, any mutation with a selective coefficient *s* much greater than 1/*N*_*e*_ is likely to rise in frequency by selection provided it becomes established in the population. By establishment (Table 1), we mean the mutant must also be lucky enough to reach a frequency where it escapes genetic drift and then is guided deterministically by selection (Fig. 1). This frequency is typically 1/*s* individuals (2, 43). Thus, a fairly typical big-benefit mutation that increases fitness by 10% (*s* ~ 0.1) (19, 20) exceeds the general *N_e_s* threshold by 4 orders of magnitude. If its population size reaches a mere 10 individuals, it is nearly guaranteed to be governed by selection and is likely to completely take over (fix) the population in the absence of competition. However, should this big-benefit mutation cooccur in a population with an even more beneficial mutation (*s* = 0.2 or more), it may lose out to this superior competitor by the process of clonal interference (Table 1 and Fig. 2). In fact, even though beneficial mutations may amount to only 1% of all mutations (or less), large populations guarantee that many beneficial mutations will arise and compete under these conditions, with only the best of them ever reaching a detectable frequency of 0.01 or more (Fig. 2) (2, 19, 21).

As the experiment progresses, suppose mutants with some potentially adaptive phenotype (a change in colony morphology indicative of altered secretions, growth parameter, metabolic capacity, host colonization, etc.) are observed in a population after seven transfers or ~46 generations. You pick these mutants to subculture them, and their phenotypes appear heritable. What is the probability that these mutants involve adaptations driven by selection and did not become frequent by the random forces of mutation and drift? Virtually certain. Here is why: any given mutation with no effect on fitness (and likely no phenotype) will be governed by genetic drift and will reach a potentially detected frequency of 0.1 in the following number of generations, where *g* is the number of generations, *p* is the mutant frequency, and µ is the mutation rate (22):

$$g=\frac{-\log_{2}p}{µ}=\frac{-\log_{2}0.1}{10^{-3}}$$

This would equal 3,322 generations, or 499 days in this experimental design. This is the estimated time required for any mutation to reach a frequency of 10% by genetic drift alone. For a mutant to become detected 100 times faster than this in your evolution experiment (and this is not uncommon), the mutant must have been subject to strong positive selection because it is adaptive.

As evolution experiments become commonplace for many microbial species in a range of environments, reports of parallel mutations—even identical nucleotide changes in the same gene—are becoming more frequent (15, 23). This observation is often met with the question—is this particular site hypermutable? We need to consider three more questions. First, can a high mutation rate alone drive a mutation to high frequency? Second, what role might selection have played? Third, under what conditions would parallelism occur?

Let us suppose that this position is hypermutable, not the usual 1 × 10^−10^ mutations/bp/generation but 100-fold more mutable at 1 × 10^−8 ^mutations/bp/generation. Would a high mutation rate alone cause it to rise to high frequency in multiple populations?

Using the same equation above, a specific mutation would reach a frequency of 10% by drift alone by $\frac{-\log_{2}0.1}{10^{{}^{8}}}$ = 33,219,289 generations, which is clearly implausible, so mutation pressure alone cannot lead to parallelism. However, let us imagine this mutant is strongly selected with *s* = 0.2, which is much greater than the mutation rate. It will rise to 10% frequency in $\frac{-\log_{2}0.1}{0.2}$ = 16.6 generations, or less than 3 transfer days, which is fast! Selection can cause a highly beneficial mutation to sweep very rapidly, in the typical time required for an efficient laboratory genetic screen.

However, the probability that the same mutation becomes rapidly detected in another population depends on whether this same mutation actually occurs in time. The evolution experiment we have outlined here generates ~10^5^ new mutations per day, with only 10^3^ on average surviving each bottleneck. The probability of this same mutation even recurring is the approximate per-site mutation rate of 2 × 10^−10^, which will occur on average in 10^8^ cell divisions/day ≈ 39 days or 263 generations. Site-specific parallelism is therefore still highly improbable. However, strongly selected mutations are much more likely to survive the bottleneck and may also become enriched both by demography (becoming slightly more common in the transfer by chance). Estimating the exact probability of parallelism is not straightforward because it depends on these chance effects as well as on the distribution of effects of other selected mutations within the population, but in essence, if the same mutation does indeed occur by chance in another population and escapes drift and outcompetes other mutants, it will also rapidly reach this frequency of 0.1.

To sum up, any single mutation rising from a frequency of 10^−7^ to 10^−1^ in an evolution experiment lasting weeks or months (100 to 1,000 generations) must be under positive selection, and equally important, the contribution of other population genetic processes is negligible. Parallelism at the level of nucleotides, amino acids, or even genes in replicate populations adds overwhelming evidence of strong selection on that target.

Why are these inferences useful? Given that the force of selection dominates evolution experiments conducted in large populations, mutants rising to high frequency must have acquired adaptive traits in the selective environment. Furthermore, the large population sizes typically used in these experiments often generate multiple beneficial mutants that compete with one another (Fig. 2), meaning that those that ultimately become detectable (e.g., as colonies on a plate) are among the most fit available. Genome sequencing of these mutants can therefore be used as a forward genetic screen for traits that enhance fitness in any environment, including new host organisms (9, 15, 24–27). Sequencing multiple mutants from independent populations or diverse samples of the evolved populations can expand the sample of adaptive mutations. Any parallelism at the level of gene or pathway provides powerful inference that alterations in this pathway provide an ideal adaptive solution. As one example from our screen of adaptations to biofilm growth, we observed four independent D652N mutations in the response regulator protein WspE in different populations of Burkholderia cenocepacia selected for only 32 to 64 generations (15). These mutants produced a characteristic wrinkly colony morphology and increased biofilm. No specific features of the nucleotide sequence indicate that this site is hypermutable, but this remarkable parallelism is consistent with the fitness advantage of *s* ≈ 1.0 that rapidly displaces the ancestor (15). These results also demonstrate that this aspartate residue (and not the adjacent aspartate at position 653) that is outside the primary receiver domain is functionally important for signaling that the cell is in contact with the surface (28, 44). Another example of an evolution experiment serving as a genetic screen involved E. coli selected for growth in test tubes in minimal medium containing glucose (19). Some of the most fit mutants acquired partial loss-of-function mutations in *yfgA* and *prc*, genes that encode a cytoskeletal protein and a protease involved in peptidoglycan synthesis. We reasoned that both mutations could result in the synthesis of a more limited peptidoglycan layer to facilitate transport but also alter cell shape, which we indeed observed as the production of more spherical cells (19). Put simply, this evolve-and-resequence approach is likely to find the best mutations (or mutation combinations) to respond to any selective challenge and can inform how the affected proteins function and enable the mutant to outcompete its ancestor.

Researchers with diverse interests have recognized this opportunity, leading to an explosion of the kinds of questions addressed by experimental evolution and two successful American Society for Microbiology (ASM) meetings focused on the subject. For example, experimental evolution has been used to discover: (i) how bacteria evolve resistance to antibiotics (29, 30) or environmental pollutants (31), (ii) how microbes evolve mutualistic or antagonistic social interactions with other microbes (32–35), (iii) how bacteriophage undergo host range expansion (36, 37), (iv) how microbes adapt to current hosts (38) or novel hosts (9), and (vi) how pathogens evolve during infections (39). These imaginative studies have identified new pathways that reveal how these microbes adapt to new conditions and some recurring themes, such as the selection for mutations in regulators that produce multiple adaptive traits at once. With the wealth of knowledge and experience from almost 150 years of evolution experiments and an influx of creative young investigators, the future of using evolution experiments to reveal unknown mechanisms of adaptation could not be brighter.

## References

[1] Good BH, McDonald MJ, Barrick JE, Lenski RE, Desai MM 2017 The dynamics of molecular evolution over 60,000 generations. Nature 551:45–50. doi:10.1038/nature24287. 29045390 PMC5788700

[2] Levy SF, Blundell JR, Venkataram S, Petrov DA, Fisher DS, Sherlock G 2015 Quantitative evolutionary dynamics using high-resolution lineage tracking. Nature 519:181–186. doi:10.1038/nature14279. 25731169 PMC4426284

[3] O’Malley MA, Travisano M, Velicer GJ, Bolker JA 2015 How do microbial populations and communities function as model systems? Q Rev Biol 90:269–293. doi:10.1086/682588. 26591851

[4] Bull JJ 2015 Evolutionary reversion of live viral vaccines: can genetic engineering subdue it? Virus Evol 1:vev005. doi:10.1093/ve/vev005. 27034780 PMC4811365

[5] Lenski RE 2017 Experimental evolution and the dynamics of adaptation and genome evolution in microbial populations. ISME J 11:2181–2194. doi:10.1038/ismej.2017.69. 28509909 PMC5607360

[6] Cooper VS, Schneider D, Blot M, Lenski RE 2001 Mechanisms causing rapid and parallel losses of ribose catabolism in evolving populations of *Escherichia coli* B. J Bacteriol 183:2834–2841. doi:10.1128/JB.183.9.2834-2841.2001. 11292803 PMC99500

[7] Cooper TF, Rozen DE, Lenski RE 2003 Parallel changes in gene expression after 20,000 generations of evolution in *Escherichia coli*. Proc Natl Acad Sci U S A 100:1072–1077. doi:10.1073/pnas.0334340100. 12538876 PMC298728

[8] Schneider D, Duperchy E, Coursange E, Lenski RE, Blot M 2000 Long-term experimental evolution in Escherichia coli. IX. Characterization of insertion sequence-mediated mutations and rearrangements. Genetics 156:477–488. 11014799 10.1093/genetics/156.2.477 PMC1461276

[9] Pankey MS, Foxall RL, Ster IM, Perry LA, Schuster BM, Donner RA, Coyle M, Cooper VS, Whistler CA 2017 Host-selected mutations converging on a global regulator drive an adaptive leap by bacteria to symbiosis. Elife 6:e24414. doi:10.7554/eLife.24414. 28447935 PMC5466423

[10] Gerrish PJ, Lenski RE 1998 The fate of competing beneficial mutations in an asexual population. Genetica 102-103:127–144. doi:10.1007/978-94-011-5210-5_12. 9720276

[11] Dillon MM, Sung W, Sebra R, Lynch M, Cooper VS 2017 Genome-wide biases in the rate and molecular spectrum of spontaneous mutations in Vibrio cholerae and Vibrio fischeri. Mol Biol Evol 34:93–109. doi:10.1093/molbev/msw224. 27744412 PMC5854121

[12] Lee H, Popodi E, Tang HX, Foster PL 2012 Rate and molecular spectrum of spontaneous mutations in the bacterium Escherichia coli as determined by whole-genome sequencing. Proc Natl Acad Sci U S A 109:E2774–E2783. doi:10.1073/pnas.1210309109. PMC3478608 22991466

[13] Cottinet D, Condamine F, Bremond N, Griffiths AD, Rainey PB, de Visser JAGM, Baudry J, Bibette J 2016 Lineage tracking for probing heritable phenotypes at single-cell resolution. PLoS One 11:e0152395. doi:10.1371/journal.pone.0152395. 27077662 PMC4831777

[14] van der Woude MW, Bäumler AJ 2004 Phase and antigenic variation in bacteria. Clin Microbiol Rev 17:581–611. doi:10.1128/CMR.17.3.581-611.2004. 15258095 PMC452554

[15] Cooper VS, Staples RK, Traverse CC, Ellis CN 2014 Parallel evolution of small colony variants in Burkholderia cenocepacia biofilms. Genomics 104:447–452. doi:10.1016/j.ygeno.2014.09.007. 25263109

[16] Luria SE, Delbrück M 1943 Mutations of bacteria from virus sensitivity to virus resistance. Genetics 28:491–511. 17247100 10.1093/genetics/28.6.491 PMC1209226

[17] Martin P, van de Ven T, Mouchel N, Jeffries AC, Hood DW, Moxon ER 2003 Experimentally revised repertoire of putative contingency loci in Neisseria meningitidis strain MC58: evidence for a novel mechanism of phase variation. Mol Microbiol 50:245–257. doi:10.1046/j.1365-2958.2003.03678.x. 14507378

[18] Gallie J, Libby E, Bertels F, Remigi P, Jendresen CB, Ferguson GC, Desprat N, Buffing MF, Sauer U, Beaumont HJE, Martinussen J, Kilstrup M, Rainey PB 2015 Bistability in a metabolic network underpins the de novo evolution of colony switching in Pseudomonas fluorescens. PLoS Biol 13:e1002109. doi:10.1371/journal.pbio.1002109. 25763575 PMC4357382

[19] Dillon MM, Rouillard NP, Van Dam B, Gallet R, Cooper VS 2016 Diverse phenotypic and genetic responses to short-term selection in evolving Escherichia coli populations. Evolution 70:586–599. doi:10.1111/evo.12868. 26995338

[20] Peng F, Widmann S, Wünsche A, Duan K, Donovan KA, Dobson RCJ, Lenski RE, Cooper TF 2018 Effects of beneficial mutations in pykF gene vary over time and across replicate populations in a long-term experiment with bacteria. Mol Biol Evol 35:202–210. doi:10.1093/molbev/msx279. 29069429 PMC5850340

[21] Patwa Z, Wahl LM 2008 The fixation probability of beneficial mutations. J R Soc Interface 5:1279–1289. doi:10.1098/rsif.2008.0248. 18664425 PMC2607448

[22] Lenski RE, Rose MR, Simpson SC, Tadler SC 1991 Long-term experimental evolution in Escherichia coli. I. Adaptation and divergence during 2,000 generations. Am Nat 138:1315–1341. doi:10.1086/285289.

[23] Lind PA, Farr AD, Rainey PB 2017 Evolutionary convergence in experimental Pseudomonas populations. ISME J 11:589–600. doi:10.1038/ismej.2016.157. 27911438 PMC5322309

[24] O’Rourke D, Fitzgerald CE, Traverse CC, Cooper VS 2015 There and back again: consequences of biofilm specialization under selection for dispersal. Front Genet 6:18. doi:10.3389/fgene.2015.00018. 25717335 PMC4324302

[25] Lind PA, Farr AD, Rainey PB 2015 Experimental evolution reveals hidden diversity in evolutionary pathways. Elife 4:e07074. doi:10.7554/eLife.07074. PMC4395868 25806684

[26] McDonald MJ, Gehrig SM, Meintjes PL, Zhang X-X, Rainey PB 2009 Adaptive divergence in experimental populations of *Pseudomonas fluorescens*. IV. Genetic constraints guide evolutionary trajectories in a parallel adaptive radiation. Genetics 183:1041–1053. doi:10.1534/genetics.109.107110. 19704015 PMC2778958

[27] Marchetti M, Capela D, Glew M, Cruveiller S, Chane-Woon-Ming B, Gris C, Timmers T, Poinsot V, Gilbert LB, Heeb P, Médigue C, Batut J, Masson-Boivin C 2010 Experimental evolution of a plant pathogen into a legume symbiont. PLoS Biol 8:e1000280. doi:10.1371/journal.pbio.1000280. 20084095 PMC2796954

[28] O’Connor JR, Kuwada NJ, Huangyutitham V, Wiggins PA, Harwood CS 2012 Surface sensing and lateral subcellular localization of WspA, the receptor in a chemosensory-like system leading to c-di-GMP production. Mol Microbiol 86:720–729. doi:10.1111/mmi.12013. 22957788 PMC3501340

[29] Toprak E, Veres A, Michel J-B, Chait R, Hartl DL, Kishony R 2011 Evolutionary paths to antibiotic resistance under dynamically sustained drug selection. Nat Genet 44:101–105. doi:10.1038/ng.1034. 22179135 PMC3534735

[30] Barbosa C, Trebosc V, Kemmer C, Rosenstiel P, Beardmore R, Schulenburg H, Jansen G 2017 Alternative evolutionary paths to bacterial antibiotic resistance cause distinct collateral effects. Mol Biol Evol 34:2229–2244. doi:10.1093/molbev/msx158. 28541480 PMC5850482

[31] Zhou A, Lau R, Baran R, Ma J, von Netzer F, Shi W, Gorman-Lewis D, Kempher ML, He Z, Qin Y, Shi Z, Zane GM, Wu L, Bowen BP, Northen TR, Hillesland KL, Stahl DA, Wall JD, Arkin AP, Zhou J 2017 Key metabolites and mechanistic changes for salt tolerance in an experimentally evolved sulfate-reducing bacterium, Desulfovibrio vulgaris. mBio 8:e01780-17. doi:10.1128/mBio.01780-17. 29138306 PMC5686539

[32] McCully LM, Bitzer AS, Seaton SC, Smith LM, Silby MW 2018 Social motility: interaction between two sessile soil bacteria leads to emergence of surface motility. bioRxiv https://doi.org/10.1101/296814. 10.1128/mSphere.00696-18 PMC6354810 30700513

[33] Martin M, Hölscher T, Dragoš A, Cooper VS, Kovács ÁT 2016 Laboratory evolution of microbial interactions in bacterial biofilms. J Bacteriol 198:2564–2571. doi:10.1128/JB.01018-15. 27044625 PMC5019067

[34] Martin M, Dragoš A, Hölscher T, Maróti G, Bálint B, Westermann M, Kovács ÁT 2017 *De novo* evolved interference competition promotes the spread of biofilm defectors. Nat Commun 8:15127. doi:10.1038/ncomms15127. 28462927 PMC5418572

[35] Hillesland KL, Stahl DA 2010 Rapid evolution of stability and productivity at the origin of a microbial mutualism. Proc Natl Acad Sci U S A 107:2124–2129. doi:10.1073/pnas.0908456107. 20133857 PMC2836651

[36] Burmeister AR, Lenski RE, Meyer JR 2016 Host coevolution alters the adaptive landscape of a virus. Proc Biol Sci 283:20161528. doi:10.1098/rspb.2016.1528. PMC5046904 27683370

[37] Shapiro JW, Turner PE 2018 Evolution of mutualism from parasitism in experimental virus populations. Evolution 72:707–712. doi:10.1111/evo.13440. 29380361

[38] Guidot A, Jiang W, Ferdy J-B, Thébaud C, Barberis P, Gouzy J, Genin S 2014 Multihost experimental evolution of the pathogen Ralstonia solanacearum unveils genes involved in adaptation to plants. Mol Biol Evol 31:2913–2928. doi:10.1093/molbev/msu229. 25086002

[39] King KC, Brockhurst MA, Vasieva O, Paterson S, Betts A, Ford SA, Frost CL, Horsburgh MJ, Haldenby S, Hurst GD 2016 Rapid evolution of microbe-mediated protection against pathogens in a worm host. ISME J 10:1915–1924. doi:10.1038/ismej.2015.259. 26978164 PMC5029159

[40] Zanini F, Neher RA 2012 FFPopSim: an efficient forward simulation package for the evolution of large populations. Bioinformatics 28:3332–3333. doi:10.1093/bioinformatics/bts633. 23097421 PMC3519462

[41] Hill WG, Robertson A 1966 The effect of linkage on limits to artificial selection. Genet Res 8:269–294. doi:10.1017/S0016672300010156. 5980116

[42] Dillon MM, Cooper VS 2016 The fitness effects of spontaneous mutations nearly unseen by selection in a bacterium with multiple chromosomes. Genetics 204:1225–1238. doi:10.1534/genetics.116.193060. 27672096 PMC5105853

[43] Desai MM, Fisher DS 2007 Beneficial mutation selection balance and the effect of linkage on positive selection. Genetics 176:1759–1798. doi:10.1534/genetics.106.067678. 17483432 PMC1931526

[44] Kim W, Levy SB, Foster KR 2016 Rapid radiation in bacteria leads to a division of labour. Nat Commun 7:10508. doi:10.1038/ncomms10508. 26852925 PMC4748119

